# Zymo-Parts: A Golden Gate Modular Cloning Toolbox for Heterologous Gene Expression in *Zymomonas mobilis*

**DOI:** 10.1021/acssynbio.2c00428

**Published:** 2022-11-08

**Authors:** Gerrich Behrendt, Jonas Frohwitter, Maria Vlachonikolou, Steffen Klamt, Katja Bettenbrock

**Affiliations:** Analysis and Redesign of Biological Networks, Max Planck Institute for Dynamics of Complex Technical Systems, Sandtorstr. 1, 39106 Magdeburg, Germany

**Keywords:** *Zymomonas mobilis*, genome engineering, lactate production, modular cloning, Golden-Gate cloning, promoter study

## Abstract

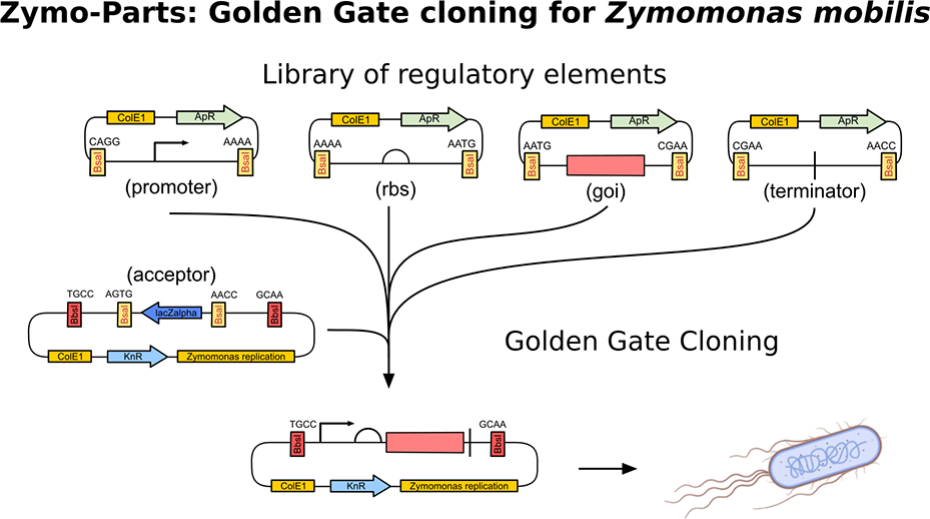

*Zymomonas mobilis* is a microorganism with extremely high sugar consumption and ethanol production rates and is generally considered to hold great potential for biotechnological applications. However, its genetic engineering is still difficult, hampering the efficient construction of genetically modified strains. In this work, we present Zymo-Parts, a modular toolbox based on Golden-Gate cloning offering a collection of promoters (including native, inducible, and synthetic constitutive promoters of varying strength), an array of terminators and several synthetic ribosomal binding sites and reporter genes. All these parts can be combined in an efficient and flexible way to achieve a desired level of gene expression, either from plasmids or via genome integration. Use of the GoldenBraid-based system also enables an assembly of operons consisting of up to five genes. We present the basic structure of the Zymo-Parts cloning system, characterize several constitutive and inducible promoters, and exemplify the construction of an operon and of chromosomal integration of a reporter gene. Finally, we demonstrate the power and utility of the Zymo-Parts toolbox for metabolic engineering applications by overexpressing a heterologous gene encoding for the lactate dehydrogenase of *Escherichia coli* to achieve different levels of lactate production in *Z. mobilis*.

## Introduction

*Zymomonas mobilis* is a facultative anaerobic, Gram-negative alphaproteobacterium and one of the best natural ethanol producers from glucose with extremely high ethanol yield (up to 97% of the maximum yield) and ethanol production rates.^1^ It uses the Entner–Doudoroff pathway for sugar breakdown, which allows for very high fluxes resulting in extraordinary glucose uptake rates exceeding that of *Escherichia coli* or yeast by a factor of 3–4.^2^*Z. mobilis* is also considered as a potential platform organism for production of other bulk chemicals, especially those lying downstream of pyruvate. However, three key aspects currently hamper routine use of *Z. mobilis* in biotechnological production processes. First, its native substrate range is limited to glucose, sucrose, and fructose.^3^ Second, it has only a low tolerance toward inhibitory substances, arising, for example, from treatment of lignocellulosic biomass.^4^ Lastly, despite some recent developments to expand the extraordinary capacity for ethanol synthesis toward other products such as lactate,^5^ isobutanol,^5,6^ poly-3-hydroxybutyrate,^7^ and 2,3-butanediol,^8^ there is an increasing need for efficient genetic tools for metabolic engineering.

Significant research efforts in the past strived to develop efficient genetic tools for *Z. mobilis.* Several studies focused on shuttle vectors built from native plasmids of *Z. mobilis*, mostly based on plasmids of strain ATCC10988.^5,9−12^ Recently, native promoters and synthetic ribosomal binding sites were tested for their expression strength.^13^ However, to date, a comprehensive toolbox for *Z. mobilis* allowing the efficient reutilization and combination of such elements is still lacking.

In this work, we present Zymo-Parts, a modular toolbox based on Golden-Gate cloning, allowing for a variable combination of different inducible and constitutive promoters and ribosome binding sites with genes of interest into versions of a shuttle vector based on pZMOB6. Since the first publication of Golden-Gate cloning in 2009^14^ and following its adaptation for modular cloning,^15^ many related toolboxes have been published for various applications. Some toolboxes serve for larger groups of organism like plants^16^ or even unite different kingdoms of life,^17^ while others focus on single species like *E. coli*(18) or *Y. lipolytica*.^19^ The rising popularity of the Golden-Gate cloning system is based on the fact that it facilitates rapid, efficient, and directed combination of DNA modules. In this way, different genetic elements, e.g., for gene expression, can be combined, thereby enabling systematic testing of single elements or of combinations of elements. Using Golden-Gate cloning, it is possible to combine different promoters, regulators, ribosomal binding sites, and terminators into transcription units (TUs) for heterologous gene expression. It also offers easy assembly of multi-TU vectors.

The applicability of Golden Gate assembly for *Z. mobilis* has already been demonstrated,^20^ but a complete toolbox tailored for this organism is still not available. The Zymo-Parts toolbox offers a collection of native promoters, together with synthetic constitutive and inducible promoters of varying strength, an array of terminators, and several synthetic ribosomal binding sites and reporter genes. All these parts can be combined in a flexible way using Golden Gate assembly to achieve a desired level of expression. Generally, the constructs can also be used as templates for other assembly approaches like Gibson assembly or traditional cloning using type II restriction enzymes. We present the basic structure of the Zymo-Parts cloning system, characterize several constitutive and inducible promoters with regard to their performance in *Z. mobilis*, and exemplify the construction of an operon (via the GoldenBraid system) as well as the chromosomal integration of a reporter gene. Regarding the latter, homologous recombination with replacement of a specific locus by an antibiotic resistance is a commonly used method for gene deletion in *Z. mobilis*.^21,22^ Golden-Gate cloning systems allow for a modular assembly of parts and hence are perfectly suited to construct plasmid templates for homology-based recombination. We used our toolbox to assemble two constructs for recombination of an antibiotic resistance paired with either a transcription unit for *mcherry* or *ldhA* into the locus ZMO0028. Finally, as a proof-of-principle application for metabolic engineering, we overexpressed a heterologous gene encoding the lactate dehydrogenase of *E. coli* at different levels to enhance lactate production in *Z. mobilis* to varying degrees.

## Results

### Composition of the Zymo-Parts Toolbox

**Figure 1:**
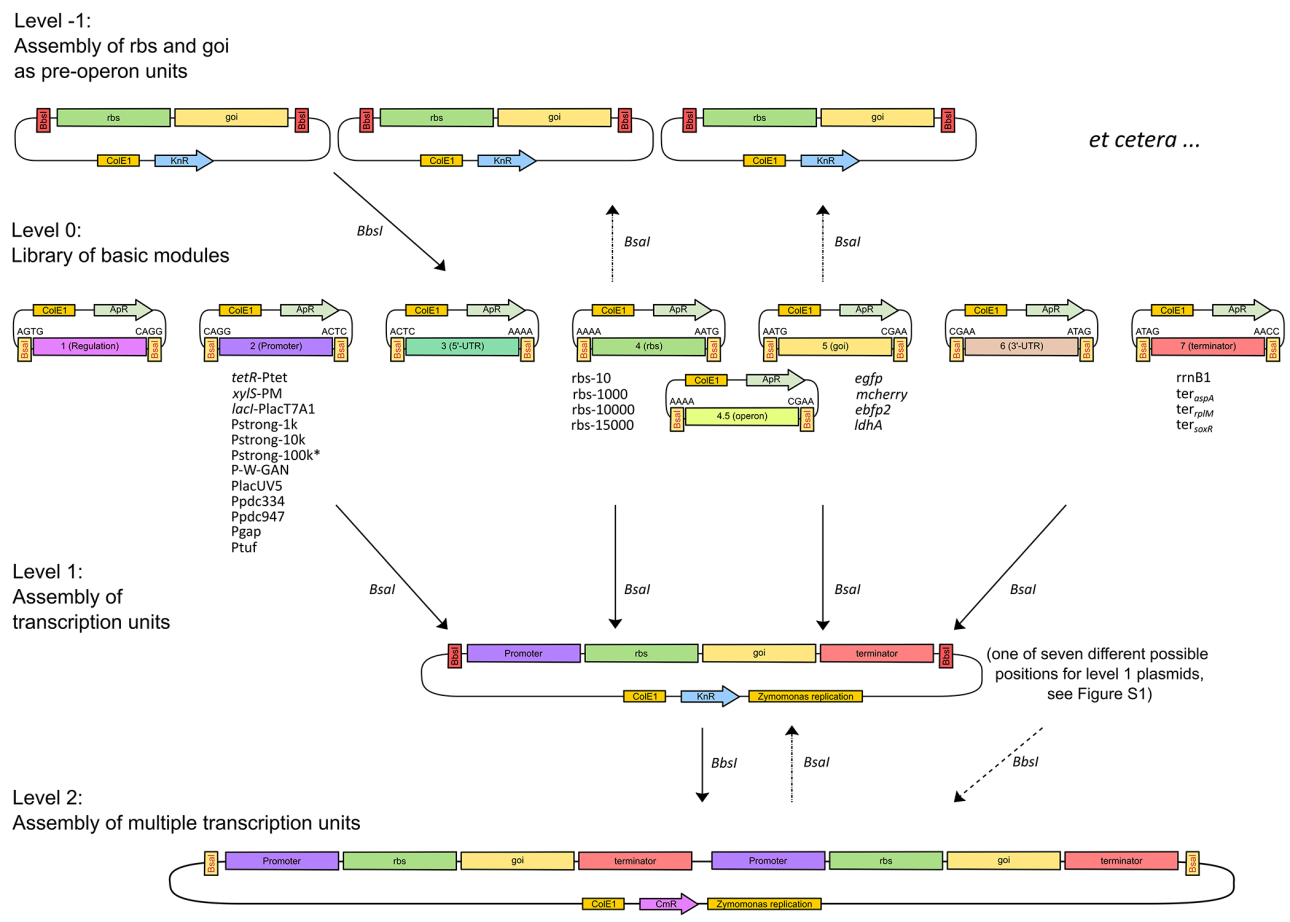
Scheme of the Zymo-Parts system. The system is organized in four levels with each level meant to carry out specific functions. Level 0 is designed to house individual, basic modules that can be assembled into level neg1 (−1) to form rbs–goi pairs or to assemble TUs in level 1. The rbs–goi pair of level neg1 can be assembled into multigene constructs in level 0, which can then be put into level 1 to form an operon as a TU. Further, the TUs of level 1 can be combined into coexpression plasmids in level 2, which can again be combined back to form even larger constructs in level 1. All this is made possible through alternating type IIS restriction enzyme (BbsI, BsaI) recognition sites between levels, as is typical for Golden-Gate cloning systems. Additionally, blue–white screening through lacZα facilitates the procedure.

The central goal of our Zymo-Parts toolbox described in the following is to offer genetic elements functional in *Z. mobilis* that can be easily and efficiently assembled into expression vectors for heterologous gene expression in *Z. mobilis.* The toolbox covers a range of promoters (P), ribosomal binding sites (rbs), and terminators (ter) as well as fluorescent reporters. We constructed Zymo-Parts based on a Golden Gate modular cloning system using four levels (Figure 1). Level negative 1 (level neg1) is designed for combining a ribosome binding site with a gene of interest (goi) into modules that can be used to assemble synthetic operons. Level 0 is used for cloning of single elements of transcription units (TU) like promoters or genes of interest as well as operons. Level 1 allows for an assembly of these elements offering seven different positions in two orientations. Finally, level 2 allows for the assembly of different TUs into multi-TU constructs or the combination of a transcription unit with other elements, like homology arms for homologous recombination into the genome of *Z. mobilis*.

GoldenBraid mechanisms were used to incorporate the back and forth between level 0 and level neg1 and between level 2 and level 1.^23^ This allows the combination of rbs and goi modules into combined modules and the construction of polycistronic TU based on these combined modules. Dummies and end-linkers are provided that enable the assembly of fewer than seven elements from level 1 into level 2 and vice versa. Level 1 and level 2 acceptor plasmids have a ColE1 origin for replication in *E. coli* and in addition the replication elements and origin of the native *Z. mobilis* ATCC10988 plasmid pZMOBP6.^9^ This plasmid has been used previously for the construction of *Zymomonas* shuttle vectors.^5,10^ The overhangs for the ligation needed for Golden-Gate cloning were chosen based on the best fidelity of four base overhangs.^24^ So far, the system is composed of more than 65 different acceptors, dummies, and end-linkers (Figure S1) but is open to future expansions.

### Selection and Testing of Constitutive Promoters

**Figure 2:**
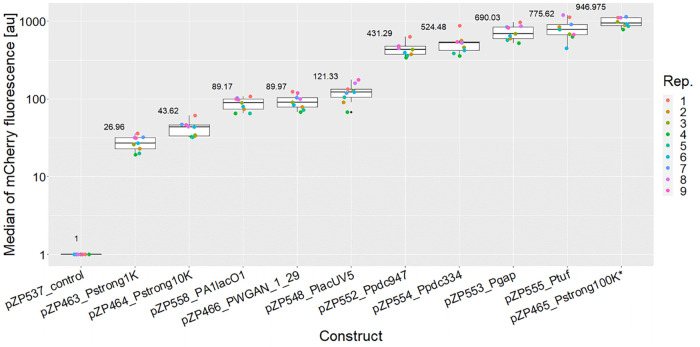
Box-plots showing the medians of mCherry fluorescence intensity driven by constitutive promoters in ZM4. In total, nine biological replicates (Rep.) from three independent cultivations were measured using flow cytometry. The median of the data points per construct is displayed in each box.

We tested four synthetic promoters (Pstrong1k, Pstrong10k, Pstrong100k*, and PWGAN-1-29^25^), four native promoters of *Z. mobilis* ZM4 (Ppdc947, Ppdc334, Pgap, and Ptuf), as well as two derivatives of the *E. coli* Plac promoter (PA1lacO1 and PlacUV5). The native promoters kept their respective rbs while the non-native promoters were paired with rbs10k.^13^ Three of the purely synthetic promoters were designed *de novo* using the promoter calculator (https://salislab.net/software/), a 346-parameter model that predicts site-specific transcription initiation rates.^26^ Version v1.0 of the promoter calculator was used, with input specifications “Escherichia coli str. K-12 substr. MG1655 (NC_000913)”, as the only available organism at the time, assuming a rather conserved σ^70^, with the optimization mode “Single-TSS Design” and with upstream and mRNA sequences of the planned plasmids pZP463–465, respectively, with targeted transcription rates of 1000, 10 000, and 100 000 au [0.063 RNAP/(DNA min)] (sequences of the promoters are given in Figure S2). In spite of several cloning attempts, the promoter version for 100 000 au (Pstrong100k) could not be assembled. Only a few colonies were obtained during assembly of this construct in *E. coli*, and all tested colonies harbored point mutations. However, a point mutated version, Pstrong100k*, was tested for *mcherry*(10) expression in *Z. mobilis* and surpassed all other promoters used in this study with respect to the median amount of mCherry fluorescence detected (Figure 2). Pstrong100k and Pstrong100k* deviate only in the Pribnow-Box (5′-TATAAT-3′ vs 5′-TATAT-3′, Figure S2). The other synthetic promoter, PWGAN-1-29, was taken from a promoter library generated by an AI-based framework for *de novo* promoter design in *E. coli*(25) and was chosen for its high transcription initiation rate. PlacUV5 was chosen as it was shown to work in *Z. mobilis* ZM4 before;^13,27^ PA1lacO1 was chosen as it will later be used for IPTG-based induction in combination with LacI (pZP547). The ten constitutive promoters were combined with the level 0 modules for *mcherry* (pZP125) and for the terminator of *soxR* (pZP289). The toolbox includes four terminators: T_*rrnB1*_, T_*aspA*_, T_*rplM*-*rpsI*_, and T_*soxR*_.^28^

All modules were assembled into level 1 position 3 reverse acceptors through cut-ligation. Constructs carrying the ten constitutive promoters were compared for their *mcherry* expression strength in *Z. mobilis* ZM4 using flow cytometry (Figure 2). All tested constitutive promoters resulted in clearly detectable mCherry fluorescence. As negative control, pZP537 was used, which is similar to pZP465 (Pstrong100k*–rbs10k–*mcherry*–TsoxR) but with *mcherry* replaced by a dummy sequence (pZP219). All nine biological replicates of the pZP537 control had a measured intensity median for mCherry fluorescence of 1.

Since we offer four terminators in Zymo-Parts, we constructed three additional versions of the *mcherry* expression plasmid pZP465 replacing T_*soxR*_ (pZP289) with the other three terminators T_*rrnB1*_ (pZP084), T_*aspA*_ (pZP287), and T_*rplM*-*rpsI*_ (pZP288), resulting in plasmids pZP1001, pZP1002, and pZP1003, respectively. Those three additional plasmids were tested for relative mCherry fluorescence in ZM4. All plasmids achieved similar levels of mCherry fluorescence, except for pZP1001 with T_*rrnB1*_ which generated lower fluorescence intensity (Figure S3).

### Selection and Testing of Inducible Promoters

**Figure 3:**
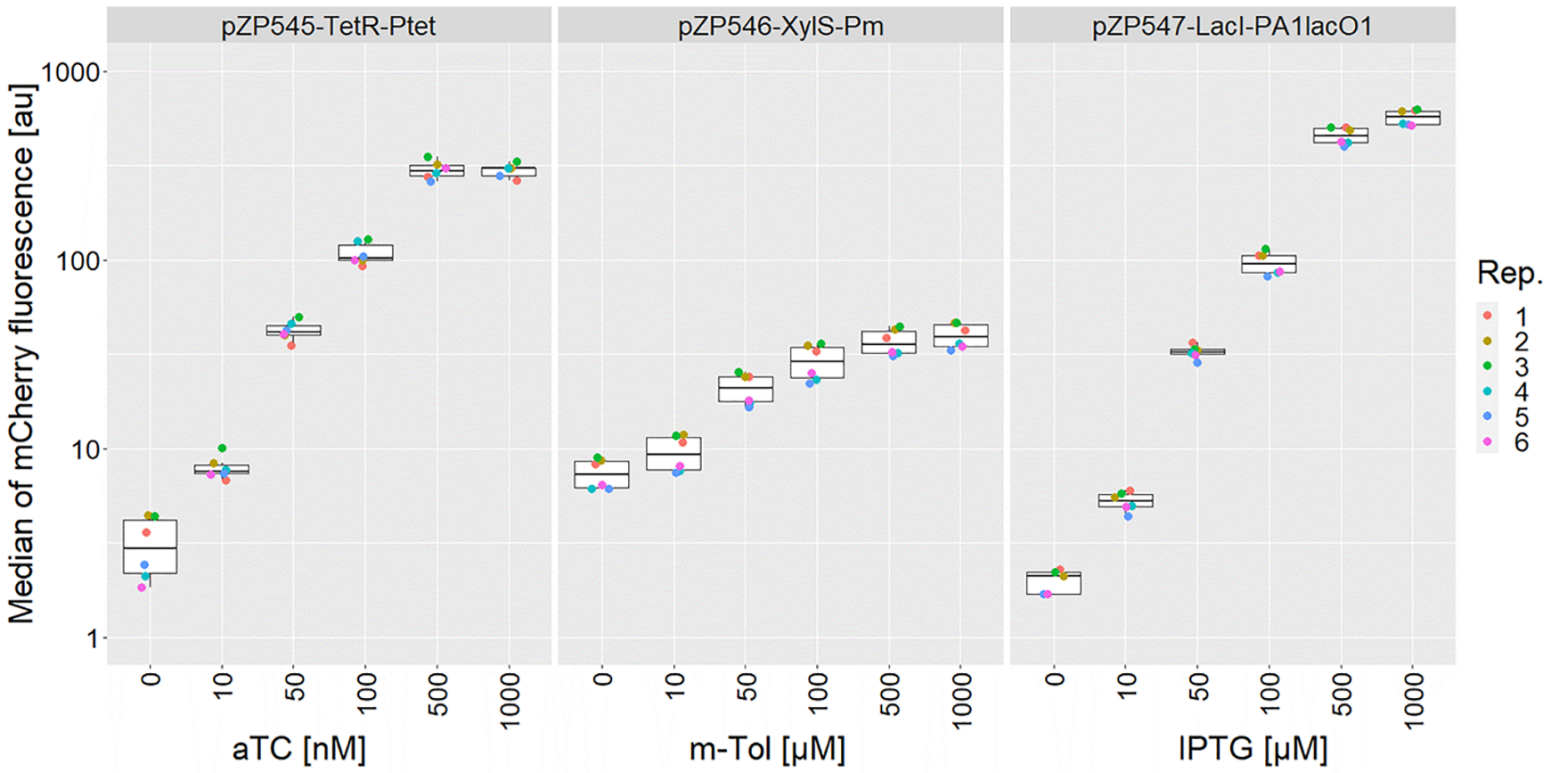
Box-plots showing the mCherry fluorescence intensity from measurements of the inducible promoters TetR-Ptet, XylS-*Pm*, and LacI-PA1lacO1 in ZM4. The induction was carried out with 10–1000 nM aTc for pZP545, 10–1000 μM *m*-Tol for pZP546, and 10–1000 μM IPTG for pZP547 as indicated in the figure. Six biological replicates (Rep.) were considered for each promoter. A negative control without *mcherry* expression corresponds to a fluorescence level of 1.

In addition to the constitutive promoters, three inducible systems were tested with respect to expression strength and regulation. The tetracycline controlled TetR-Ptet system and the IPTG controlled LacI-PA1lacO1 system had already been applied for heterologous gene expression in *Z. mobilis* ZM4.^5,10,13^ To our knowledge, the XylS-*Pm* system (variant ML1-17),^29^ regulated by *m*-toluate (*m*-Tol), has never been tested before for its functionality in *Zymomonas*. The TetR-Ptet (pZP156), XylS-*Pm* (pZP284), and LacI-PA1lacO1 systems were each assembled together with rbs10k (pZP159), *mcherry* (pZP125), and the terminator of soxR (pZP289) into level 1 position 3 reverse acceptors. The resulting plasmids (pZP545, pZP546, and pZP547, respectively) were compared regarding their *mcherry* expression levels under different inducer concentrations in *Z. mobilis* ZM4 (Figure 3). For induction of the TetR-Ptet system, anhydrotetracycline (aTc) was applied at concentrations between 10 nM and 1 μM. *m*-Tol and IPTG for induction of XylS-*Pm* and LacI-PA1lacO1, respectively, were tested at concentrations ranging from 10 μM to 1 mM.

For all three promoter systems, an inducer-dependent expression pattern was observed. Expression from TetR-Ptet was low without induction and reached a strong peak expression at 500 nM aTc. A similar behavior was observed for the LacI-PA1lacO1 with an even higher fully induced expression level reached at the highest IPTG concentration tested. Both expression systems allow for gradual expression with a broad dynamic range within 10–500 nM aTc and 10–500 μM IPTG, respectively. The observed maximal expression level of both systems is comparable to strong constitutive promoters like Ppdc. The XylS-*Pm* system also exhibits a monotonic increase with rising inducer concentrations but shows a relatively high basal expression level combined with overall low expression levels after induction and hence a lower dynamic range.

### Example Application: Lactate Production with *Z. mobilis*

**Figure 4:**
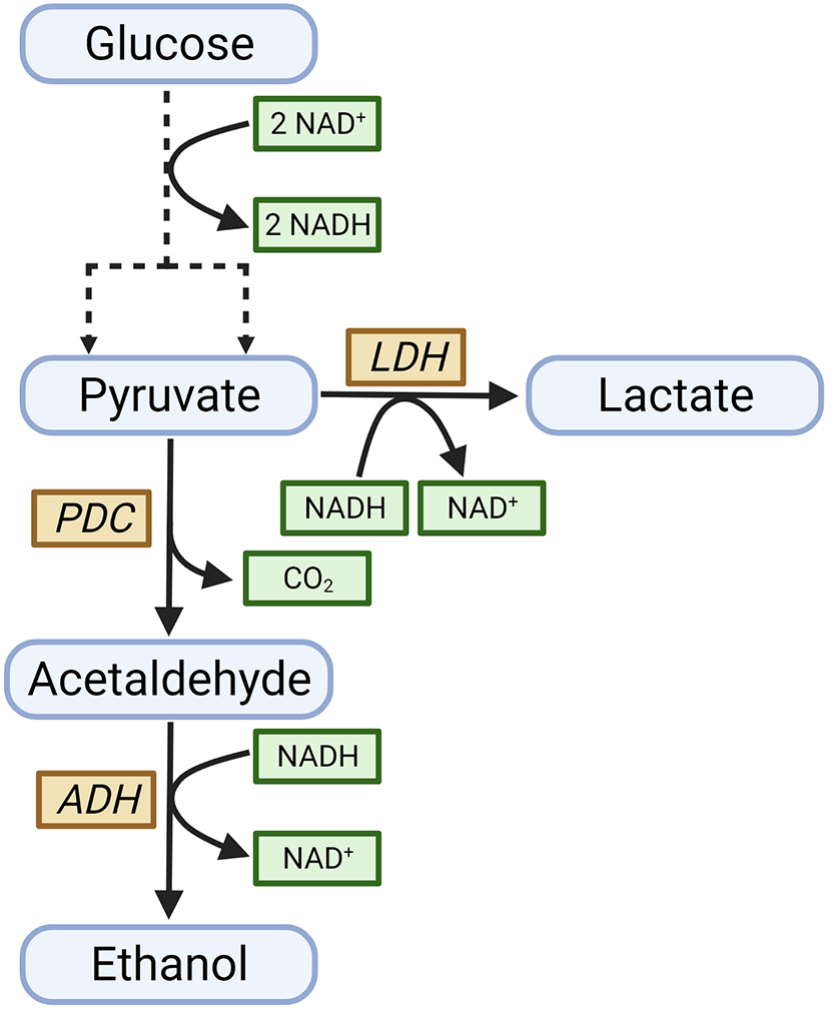
Metabolic reactions of ethanol and lactate production in *Z. mobilis*. PDC, pyruvate decarboxylase; LDH, lactate dehydrogenase; ADH, alcohol dehydrogenase.

To test our constructs for utility in a metabolic engineering scenario, we aimed to enhance lactate production in Z. *mobilis* ZM4 wild type by expressing the lactate dehydrogenase of *E. coli* from different inducible and constitutive promotors. As shown in Figure 4, production of lactate has the same redox balance as ethanol and could thus enable redirection of metabolic flux from the “metabolic highway” of *Z. mobilis* to lactate.^5^

We constructed different strains carrying plasmids with the *ldhA* gene expressed from selected constitutive and inducible promoters and determined their lactate production capabilities in anaerobic cultivations (Table 1). The construct with the inducible TetR-Ptet led to a relatively low titer of 35 mM of lactate when induced with 216 nM aTc, while using 500 nM aTc resulted in even slightly lower lactate yields, probably due to toxic effects of this higher inducer concentration. To test this, we conducted a growth experiment with varying concentrations of aTc (0–1000 nM) and could observe a reduced growth rate and final OD_600_ already at concentrations of 50 nM aTc (data not shown). Expression of *ldhA* under the control of the constitutive PlacUV5 and Pstrong100k* resulted in 81 and 86 mM lactate, respectively, with the latter representing the maximum titer of all strains and conditions studied. To test whether the LacI-PA1lacO1 system allows production of lactate with levels dependent on the inducer concentration, we tested strain pZP561, expressing *ldhA* under the control of the IPTG-inducible PA1lacO1 promotor, with varying amounts of IPTG. Indeed, we found that lactate production titers correlated with the added IPTG concentration (0 and 500 μM IPTG), similar to the results from *mcherry* expression. This demonstrates the applicability of the expression systems also for metabolic engineering purposes. Further data regarding lactate producing strains are summarized in Table S1. As expected, we observed a negative correlation between ethanol production rates/titers and those of lactate. We also found an only slight decrease in the growth rate and glucose consumption rate of lactate producing strains compared to the wild-type control strain.

**Table 1:** Lactate Production in *Z. mobilis* ZM4 Using Different Inducible and Constitutive Promotors^a^

strain	promoter	inducer	titer [mM lactate]	lactate yield [mol/mol glucose]
WT			<1	0
pZP537 empty vector	Pstrong100k*	constitutive	<1	0
pZP561	PA1lacO1	uninduced	19.6 ± 0.6	0.09
pZP561	PA1lacO1	10 μM IPTG	42.3 ± 4.8	0.19
pZP561	PA1lacO1	100 μM IPTG	70.6 ± 3.7	0.33
pZP561	PA1lacO1	500 μM IPTG	85.2 ± 2.4	0.40
pZP561	PA1lacO1	1 mM IPTG	83.1 ± 0.9	0.38
pZP255	Ptet	216 nM aTc	35.0 ± 1.7	0.17
pZP255	Ptet	500 nM aTc	29.0 ± 1.9	0.14
pZP374	PlacUV5	constitutive	81.0 ± 2.6	0.38
pZP536	Pstrong100k*	constitutive	86.0 ± 4.0	0.41
ΔZMO0028::ldhA	Pstrong100k*	constitutive	43.1 ± 0.6	0.21

a The detection limit of lactate is about 1 mM.

### Insertion of Expression Units into Chromosomal Locus ZMO0028

Since homologous recombination is frequently used for genome engineering in *Z. mobilis*, we describe in the following the utility of the Zymo-Parts toolbox for the construction of templates for homologous recombination. Locus ZMO0028, which codes for a restriction-modification system, was chosen as a target as its deletion has been reported multiple times in the literature^30−32^ and is not expected to impact growth.^33^ We created two homologous sequences for the regions upstream and downstream of the locus ZMO0028. These homology arms were positioned in level 1 pos 2 (upstream homology arm, pZP059) and level 1 pos 5 (downstream homology arm, pZP236). Furthermore, a resistance cassette for kanamycin was placed into level 1 pos 3 (pZP132). The elements were combined into the acceptor plasmid (pZP137) together with either the expression unit for *mcherry* in level 1 pos 4 (pZP597) or the expression unit for *ldhA* in level 1 pos 4 (pZP536), a dummy for level 1 pos 1 (pZP027), and the end linker for level 1 pos 5 (pZP038). The final editing plasmids (pZP778 for *mcherry* and pZP1013 for *ldhA*), which are able to replicate in *E. coli* but not in *Z. mobilis*, were transformed into ZM4 by electroporation, and the cells were plated on ZM plates harboring kanamycin.

**Figure 5:**
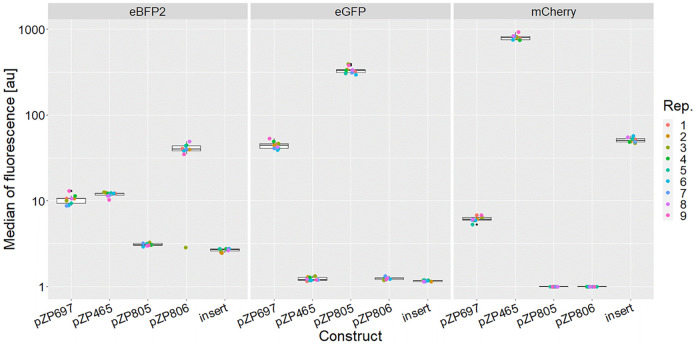
Box-plots showing the flow-cytometer-based intensity measurements for the operon construct (pZP697) and the controls: pZP465 (expressing *mcherry*), pZP805 (expressing *egfp*), and pZP806 (expressing *ebfp2*), as well as the mCherry fluorescence based on the insertion of an expression unit into chromosomal locus ZMO0028. Each dot represents the median of expression from one measurement. Nine biological replicates (Rep.) from each construct were analyzed split over two independent cultivations.

The resulting colonies after transformation with pZP778 were checked under blue light for mCherry fluorescence. All colonies showed fluorescence, and nine colonies were checked for expression strength (see Figure 5). The level of expression was much lower than for plasmid-based expression of the comparable transcription unit of pZP465 but a little stronger than the expression from pZP464 (Pstrong10k, plasmid-based). The correct insertion into the chromosome was verified by PCR (Figure S4).

The colonies obtained from the transformation with pZP1013 were checked by PCR for correct insertion. The resulting strain *ΔZMO0028::ldhA* with *ldhA* of *E. coli* inserted into the chromosome was cultivated as described above and checked for lactate production. We found that the strain synthesized lactate with a final titer of 43.1 mM (Table 1), which, as expected, is lower than with plasmid-based expression but confirms successful genomic integration of *ldhA*.

### Assembly and Testing of an Operon Encoding Three Fluorescence Proteins

Our Zymo-Parts toolbox offers a GoldenBraid-based approach to combine level 0 modules of rbs and goi into a combined level neg1 module. As each of those level neg1 modules has a determined position for the reassembly into a level 0 module, the sophisticated construction of polycistronic transcription units in level 1 containing two to five of those rbs–goi building blocks becomes possible (see Figure S1).

To test this operon-building mechanism, we constructed three level neg1 plasmids that combined three different rbs, generated by the rbs calculator^34^ with a targeted translation rate of 10 000 au, with either *egfp*,^10^*mcherry*, or *ebfp2*.^35^ Those were combined as an operon into the level 0 module pZP696 and afterward together with level 0 modules for Pstrong100k* (pZP436) and the terminator of soxR (pZP289) into the level 1 backbone pZP022 (Figure S5). This plasmid for the constitutive expression of the operon was termed pZP697. The operon plasmid as well as three control plasmids expressing the individual fluorescence proteins were analyzed to check if crosstalk occurred between the different fluorescence channels. The control plasmids were pZP465 expressing *mcherry*, pZP805 expressing *egfp*, and pZP806 expressing *ebfp2*, all sharing the same regulatory elements. Unfortunately, we observed crosstalk between mCherry and eBFP2. While eGFP and mCherry fluorescence was clearly detected in all replicates of pZP697, the eBFP2 fluorescence of this construct was only low. We cannot rule out that this fluorescence is due to the crosstalk. However, *mcherry* expression from pZP697 is rather low, and therefore, we would expect a rather low crosstalk, comparable to the one observed for the strain with *mcherry* integrated into the chromosome; hence, the blue fluorescence observed for ZM4 pZP697 is for the most part due to real eBFP2 expression. In summary, the operon construct enables the expression of a set of genes, but polar effects occur (Figure 5).

Experiments were also carried out with a plasmid (pZP741) equipped with identical rbs (rbs10k) upstream of all three fluorescence genes. Here, we observed cultures showing varying levels of fluorescence for all three proteins. An exemplary comparison between the two operons can be found in Figure S6, showing the raw histograms from flow cytometry for one cultivation per construct. We speculate that pZP741 is more susceptible to recombination in *Z. mobilis*, since using rbs10k for all genes generates three 33 bp homology regions. The fluorescence observed in ZM4 pZP741 is then likely a product of recombination events resulting in subcultures with different versions of the original operon. This suggests using different rbs when building operons in *Z. mobilis.*

### Efficiency of Assembly

We finally evaluated the assembly efficiency of our toolbox. As already mentioned before, for our Golden Gate Assembly, we used the four base overhangs described by Potatov et al.,^24^ for which a high fidelity could be shown. We did not aim to exhaustively reassess the fidelity but tested the assembly efficiency for five plasmids constructed in this work: pZP536, pZP561, pZP1001, pZP1002, and pZP1003. These plasmids represent the assembly of two transcription units for *ldhA* and three transcription units for *mcherry*, with varying regulatory elements. Each of the plasmids was independently assembled through a cut-ligation three times and transformed into NEB5α. As plasmids pZP1001–1003 are all carrying a transcription unit expressing *mcherry*, they are easily testable for correct assembly by checking for red fluorescence. Further, all acceptor plasmids carry *lacZ*α that is absent in the correctly assembled plasmid and hence allow for blue–white screening. The colonies were counted, and color (white, blue, or red) was noted. The average assembly of an *mcherry* transcription unit resulted in more than 90% of red fluorescent cells and below 5% for blue and white colonies. The average assembly of the *ldhA* transcription units resulted in more than 90% white colonies (see Table S2). For pZP536 and pZP561, we randomly selected 16 white colonies and checked them for correct assembly using PCR. All tested colonies showed the correct sizes of PCR fragments, suggesting correct assembly (see Figure S7).

## Discussion

In this work, we introduce Zymo-Parts, a Golden-Gate cloning-based toolbox for modular genetic engineering of *Z. mobilis*. To the best of our knowledge, Zymo-Parts is the first comprehensive toolbox of its kind and provides a valuable platform for the genetic design of dedicated *Z. mobilis* strains. Zymo-Parts comprises a number of tested elements for plasmid-based gene expression and genome engineering, which can be easily combined, adapted, and expanded to match specific requirements.

Using the modular Golden-Gate cloning approach of Zymo-Parts, a set of plasmids could be efficiently constructed with different combinations of promoters, regulators, and rbs for constitutive and controlled gene expression in *Z. mobilis.* All constructs were tested and compared using the fluorescent protein mCherry. Overall, the selected native *Z. mobilis* promoters in combination with their original rbs showed higher levels of expression than the synthetic or heterologous promoters in combination with rbs10k, with the exception of Pstrong100k*. This was expected, as the chosen native promoters had already been described to have high expression strengths.^13^ The set of promoters contained in the Zymo-Parts toolbox allows selection for a desired expression strength ranging from low to extremely high.

In addition to natural promoters, three synthetic promoters with different expression strength were designed. Pstrong1k and Pstrong10k allowed for low to moderate expression; Pstrong100k* showed an extremely high expression strength. Notably, we were only able to obtain a mutated version of Patrong100K, with a deletion of one base in the −10 element. However, we found that the promoters designed by the promoter calculator can be ranked for expression strength in the order they were designed for, suggesting that the promoter calculator based on σ^70^ of *E. coli* can also be applied to design promoters for *Z. mobilis*.

Besides constitutive promoters, gene expression can also be controlled using inducible systems. Usage of inducible promoters allows dynamic adjustment of gene expression and broadens the applications of plasmid-based gene expression. The functionality of the TetR-Ptet and LacI-PA1lacO1 has already been shown^5^ but was not tested in a systematic way. The performance of both inducible systems is outstanding. Both promotor–regulator pairs show only a low level of uninduced gene expression but can be induced up to 100-fold. Also, a gradual induction is possible by adjustment of the inducer concentration. The third system, XylS-*Pm*, is functional in *Z. mobilis*, but its leakiness in the uninduced state as well as its rather low maximal induction leave space for optimization. Nevertheless, it might still be helpful for selected applications.

Besides expression of single genes, Zymo-Parts also supports the expression of sets of genes. We demonstrated the expression of genes of three different fluorescent reporter proteins contained in a single operon. We observed a crosstalk between mCherry and eBFP2 fluorescence that blurred the results. However, considering the low eBFP2 crosstalk signal observed for the strain with *mcherry* inserted into the chromosome, the detected blue fluorescence for the operon construct should for the most part be caused by the presence of eBFP2. As the gene for eBFP2 is in the last position of the operon, a comparatively low expression level is to be expected if polar effects occur. Notably, the fluorescence level of all three proteins is significantly lower in the operon construct than in the control strains. Given the ability of Zymo-Parts to enable systematic testing of different rbs or spacers between the genes, the construction of operons through Zymo-Parts will be further investigated in the future.

The possibility to easily build operons and combine them with regulatory systems holds great potential for metabolic engineering of *Z. mobilis.* Many attractive products targeted by different research groups require the introduction of more than one enzyme (gene); for example, isobutanol and 2,3-butanediol synthesis involve five and three enzymes, respectively.^5,10^ We demonstrated that the Zymo-Parts toolbox enables an easy and efficient assembly of operons. This facilitates the construction of plasmids for the coordinated and fine-tuned expression of a group of genes by using previously characterized elements, e.g., promoters. Zymo-Parts also allows screening of different assemblies with respect to order and ribosomal binding sites.

We could also demonstrate the utility of the Zymo-Parts system for genome engineering. The integrations of a Kan^R^ cassette and an expression unit for either *mcherry* or *ldhA* into the chromosomal locus ZMO0028 through homologous recombination with the suicide plasmids pZP778 and pZP1013 were successful. Production of mCherry from this chromosomal locus is weaker than the plasmid-based production through pZP465, using the same P and rbs; however, the expression of the chromosomal copy was stronger than the expression obtained from the second position in the operon plasmid pZP697 (see Figure 5). Similar results were obtained for the production of lactate. A chromosomal integration of a lactate dehydrogenase gene (*ldhBC* from *Bacillus coagulans*) into ZMO0028 was undertaken earlier based on a Cas12a system.^32^ This approach does not need selection for integration by antibiotics, but with the modules now available through our Zymo-Parts, any level 1 pos 4 plasmids expression unit can easily be turned into a chromosomal integration plasmid by just changing one plasmid in the assembly reaction. Site-directed integration of sequences into the chromosome of *Z. mobilis* will be greatly facilitated by a modular Zymo-Parts toolbox and will allow efficient screening for an optimal locus for stable heterologous expression of any gene or operon. Furthermore, the toolbox can be extended to feature modules for CRISPR-Cas-based genome editing as well.

We used the production of lactate to test our toolbox in a metabolic engineering application. We overexpressed the lactate dehydrogenase gene *ldhA* from *E. coli* in *Z. mobilis* ZM4, using plasmid systems as well as genomic integration, to enhance lactate production from pyruvate. By using selected inducible and constitutive promotors from our toolbox, we were able to achieve different levels of lactate production in batch cultivations (Table 1). In our strains, the yield seems to be capped at 0.4 mol of lactate per mol of glucose, and it could not be increased using stronger promoters. A knockdown of the pyruvate decarboxylase (*pdc*) with simultaneous, plasmid-borne overexpression of *ldhA* in *Z. mobilis* was shown to enable a higher lactate yield of about 0.8 mol/mol.^5^ However, this was achieved in a strain with strongly reduced PDC activity and using rich medium in contrast to our results obtained with a *pdc* WT strain cultivated in (low-cost) minimal medium.

The accessibility of Golden-Gate cloning and the availability of a library of modules will simplify metabolic engineering projects for *Z. mobilis*. The Zymo-Parts toolbox presented here, offering different elements for plasmid-based gene expression, will serve as a starting point for a more comprehensive toolbox that will collect all relevant modules for genetic engineering. In our future work, we plan to further extend our Zymo-Parts library to allow the usage of vectors utilizing different origins of replication. The toolbox will also be adapted for basic genome editing through the native type I–F CRISPR Cas system of *Z. mobilis*.

## Materials and Methods

### Strains and Media

*E. coli* NEB5α from NewEnglandBiolabs was used for replication of all plasmids and was cultivated in LB_0_ media (10 g/L tryptone, 5 g/L yeast extract, 5 g/L NaCl) at 37 °C. *Z. mobilis* strain ZM4 (ATCC 31821) was used for all experiments and cultivated in ZM (complex) medium (bacto peptone 10 g/L, yeast extract 10 g/L, glucose 20 g/L; DSMZ GmbH) at 30 °C. For growth of plasmid carrying ZM4 and of the strain with the chromosomal integration of the Kan^R^, 100 μg/mL kanamycin was added. For plasmid propagation in *E. coli*, ampicillin was added to 100 μg/mL, chloramphenicol to 25 μg/mL, and kanamycin to 50 μg/mL, respectively.

### Construction of Acceptor Plasmids and Modules

For plasmid replication in *E. coli*, we relied on the ColE1 origin of pUC19.^36^ To work with pUC19, we modified it and created two derivatives: pUC19ΔBsaIΔ*Sma*I and pUC19ΔBsaIΔlacZα. We used the plasmid lacking BsaI recognition sites as well as the *Sma*I recognition site in *lacZα* (pUC19ΔBsaIΔ*Sma*I) as a template for amplification of the *lacZα*, added flanking *Bbsi*I and *Bsa*I recognition sites by PCR, and integrated this new cloning site for our Golden-Gate cloning system into pUC19ΔBsaIΔlacZα by *Sma*I cut-ligation, using 1 μL of 10× CutSmart buffer (NEB), 1 μL of ATP (10 mM), 1 μL of acceptor plasmid (100 ng/μL), 6 μL of insert amplificate, 0.5 μL of *Sma*I (NEB), and 0.5 μL of T4 DNA ligase (NEB). The reaction was carried out at 16 °C for 12 h. For level 1 and −1 plasmids, pGB-003 (Kan^R^, ColE1 origin and replication origin of ATCC10988 pZMOB6) was used as the acceptor while, for level 2, pGB-005 (*Cm*^R^, ColE1 origin and replication origin of ATCC10988 pZMOB6) was used. A detailed description for the assembly of all acceptor plasmids can be found in the Supporting Information. A graphical overview of all basic plasmids of the cloning system is presented in Figure S1. Gene bank files of all plasmids originating from this work can be accessed at https://figshare.com/s/c0d558d0bf762ecd5e00. All plasmids were created through either blunt cloning with *Sma*I or Golden-Gate cloning with *Bbs*I or *Bsa*I. For Golden-Gate cloning, 1 μL of 10× CutSmart buffer (NEB), 1 μL of ATP (10 mM), 1 μL of acceptor plasmid (100 ng/μL), 1 μL of per donor plasmid (100 ng/μL), 0.5 μL of restriction enzyme [BbsI-HF or BsaI-HFv2 (NEB)], and 0.5 μL of T4 DNA ligase (NEB) were used; the volume was adjusted to 10 μL by adding water. For the reaction, 30 cycles of 10 min at 37 °C followed by 10 min at 16 °C were carried out, followed by a final incubation for 20 min at 37 °C. 5 μL of the cut-ligation reaction volume and 15 μL of competent NEB5α cells (NEB) were used for a transformation applying a heatshock at 42 °C for 45 s. The cells were incubated with 200 μL of LB_0_ added for 1 h before spreading either 10 or 50 μL on LB0 plates with kanamycin and 0.02 g/L 5-Brom-4-chlor-3-indoxyl-β-d-galactopyranosid (X-Gal) for blue and white screening.

The different DNA fragments to be used in the construction of the plasmids were generated by PCR using Q5 DNA polymerase (NEB) according to the recommendations of the manufacturer. The corresponding primers and assembly procedures are listed in the Supporting Information.

### Electroporation

For electroporation, a colony of *Z. mobilis* ZM4 was cultivated overnight in ZM medium. This preculture was used to inoculate a main culture to an OD_600_ of around 0.15 in ZM medium. The cultures were incubated at 30 °C without shaking in Falcon tubes. Cells were harvested by centrifugation at 4000*×g* for 10 min at 4 °C once the culture reached an OD_600_ between 0.4 and 0.7, and cells were kept on ice afterward. The pellet was washed with cold distilled water and afterward with cold 10% (w/v) glycerol. Finally, the cells were resuspended in 1/100 of the original volume in 10% (w/v) cold glycerol. Competent cells were either used directly for electroporation or stored at −80 °C. For the electroporation, 1 mm electroporation cuvettes from VWR were used together with an Easyject Prima electroporator (Equibio). The cuvettes were cooled on ice, and at least 1 μg of plasmid was mixed with 50 μL of competent ZM4. A pulse of 1800 V was applied. After electroporation, 500 μL of ZM medium was added to the cells, and the transformed cells were incubated at 30 °C for 3 h before being plated on ZM plates with the respective antibiotics.

### Flow Cytometry Analysis

For the quantification of *mcherry*, *egfp*, and *ebfp2* expression a flow cytometer was used. Single colonies were inoculated in liquid ZM medium and grown overnight at 30 °C without shaking. This preculture was used to inoculate a main culture to an OD_600_ of around 0.015 in ZM medium. After 24 h of growth, 10 μL of this main culture was diluted in 1 mL of FACS buffer (10 mM TRIS, 10 mM MgCl_2_), and the samples were analyzed with the CyFlow Space flow cytometer (Sysmex). For detection of mCherry fluorescence, a green laser (561 nm) was used for excitation, and the emission was detected using an optical filter IBP 610/30. A blue laser (488 nm) was used for excitation of eGFP, and fluorescence intensity was detected behind an IBP 527/30 optical filter. Excitation of eBFP2 was managed by a UV laser (375 nm), and fluorescence was measured using an IBP 455 filter. The data were analyzed with Flowing Software 2 (Turku Bioscience). For a comparison of the different constructs, the median relative fluorescence intensity, calculated by the aforementioned software, was determined for each measurement. For the constitutive promoter constructs, three independent experiments were carried out with three biological replicates each. For the inducible systems, two experiments were carried out with three biological replicates each. Inducer was added to the main culture at inoculation. Anhydrotetracycline (0–1 μM), isopropyl-β-d-thiogalactopyranosid (0–1 mM), and *m*-toluate (0–1 mM) were used as inducers.

### Cultivation of Lactate Production Strains

Seed cultures and batch fermentations of all lactate producing strains were performed in a *Zymomonas* minimal medium (ZMM) containing 1 g/L K_2_HPO_4_, 1 g/L KH_2_PO_4_, 0.5 g/L NaCl, 1 g/L NH_4_SO_4_, 0.2 g/L MgSO_4_·7H_2_O, 25 mg/L Na_2_MoO_4_·2H_2_O, 2.5 mg/L FeSO_4_·7H_2_O, 20 mg/L CaCl_2_·2H_2_O, 2 g/L Ca(HCO_3_)_2_, 1 mg/L calcium pantothenate, and 40 g/L glucose (modified from Jacobson et al. 2019^37^). Precultures were inoculated from cells grown on ZM media plates and cultivated in 15 mL of ZM with 100 μg/mL kanamycin overnight at 30 °C in 15 mL tubes with loosely screwed caps. Seed cultures were inoculated from the precultures to a starting OD_600_ of 0.2–0.3 after washing the cells twice with ZMM, and the cultures were grown overnight in 50 mL tubes with loosely screwed caps in 50 mL of ZMM. Anaerobic batch fermentations were performed in Infors HT Multifors 1.4 l fermenters with the following setup: 400 mL working volume, 30 °C, stirrer speed 550 rpm, 0.1 vvm aeration with 100% nitrogen, and pH 6.5 (controlled by automated addition of 1 M NaOH). Fermentation parameters were monitored using IRIS V5.3 software (Infors AG). If necessary, the respective inducers were added aseptically to the fermenters using a syringe.

### Analytics

Extracellular lactate, ethanol, and glucose were quantified by HPLC using an Agilent 1100 series system equipped with a Rezex-ROA column. 10 μL of the sample was injected onto the column by an autosampler and analyzed using isocratic elution with 4 mM H_2_SO_4_ at a flow rate of 0.5 mL/min at 60 °C and was detected with DAD and RID detectors, respectively. Quantification of glucose, ethanol, and lactate was performed using standard curves with different concentrations of the respective standards.

### PCR Test for Genome Integration

ZM4 was transformed with pZP778 to integrate expression units for *mcherry* and a Kan^R^ cassette into the chromosomal locus ZMO0028 by homologous recombination. The integration was checked by PCRs with OneTaq Quick-Load 2X master mix (NEB), used as instructed by the company. Primers were chosen to amplify either the whole locus (edited or wild type), the integrated kanamycin, or *mcherry* expression units together with the respective neighboring chromosomal region. As a template, chromosomal DNA either from wt ZM4 or from the edited strains was used. For details, see Figure S4.

## Supporting Information Available

List of all acceptors, dummies, and end-linkers made for the Zymo-Parts kit with a description of the construction process; list of all modules described in this work with a description of construction and if applicable the literature source for the genetic element; and list of all primers used in this work with their sequence (XLSX)
Further experimental data and graphical overviews of assemblies and promoter sequences (PDF)
