# Supplementary material for: Zymo-Parts: A Golden Gate Modular Cloning Toolbox for Heterologous Gene Expression in Zymomonas mobilis
Source: ACS Synth Biol. 2022 Nov 8;11(11):3855–64. doi: 10.1021/acssynbio.2c00428 (PMC9680023; doi:10.1021/acssynbio.2c00428)
Supplement: Supplementary file 2 — sb2c00428_si_002.pdf [file sb2c00428_si_002.pdf]

Supplementary File 2

Zymo-Parts: A Golden Gate Modular Cloning Toolbox for Heterologous Gene Expression in *Zymomonas mobilis*

Gerrich Behrendt[1], Jonas Frohwitter[1], Maria Vlachonikolou[1], Steffen Klamt[1], Katja Bettenbrock[1,*]

[1] Analysis and Redesign of Biological Networks, Max Planck Institute for Dynamics of Complex Technical Systems, Sandtorstr. 1, 39106 Magdeburg, Germany

* Corresponding author: bettenbrock@mpi-magdeburg.mpg.de

Content:

Figure S1: Graphical overview of the acceptors of the Z-Parts system.

Figure S2: Graphical overview of the synthetic promoter sequences created in this work.

Figure S3: mCherry fluorescence quantification of constructs with different terminators.

Figure S4: Control PCR setup and agarose gel electrophoresis for confirmation of insertions into locus ZMO0028.

Figure S5: Graphical overview of the assembly of an operon with Zymo-Parts, on the example of pZP697.

Figures S6: Comparison of exemplary fluorescence intensity histograms for operon expressing plasmids pZP697 and pZP741.

Figure S7: Agarose gel electrophoresis results of analytical PCR performed for assembly efficiency determination of pZP536 and pZP561.

Table S1: Overview of lactate production

Table S2: Colonies counted for assembly efficiency determination of pZP536, pZP561, pZP1001, pZP1002 and pZP1003.

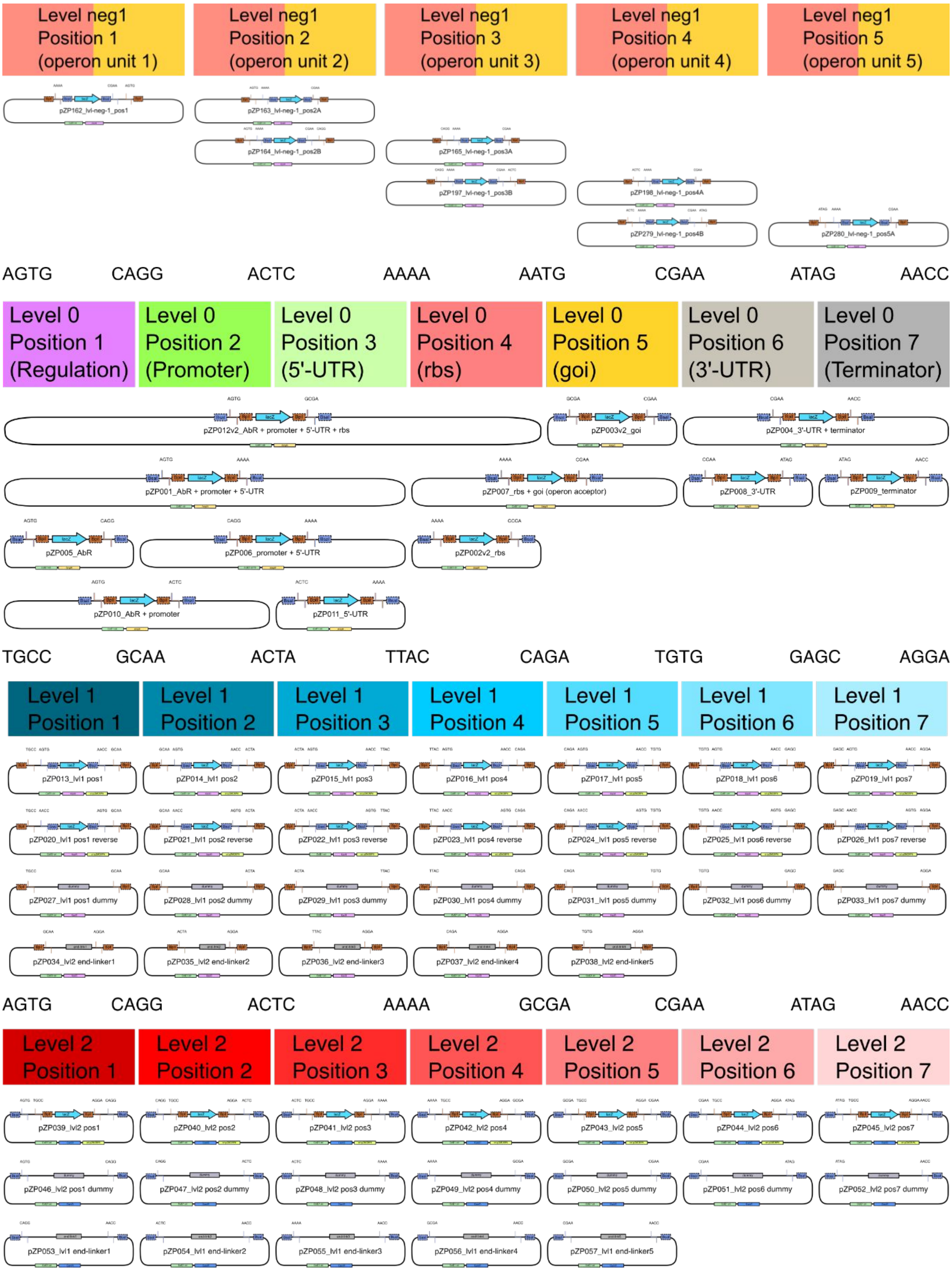

Figure S1: Graphical overview of all acceptors, dummies and end-linkers included in the Z-Parts toolbox. Each position within a level is indicated in a colored textbox above the corresponding plasmids. The overhangs used for assembly into the positions are indicated above the colored textboxes (5' to 3').

Pstrong1k

```
5´-GGTCTCaAGTGAGAATCTAGATCAAAAGAGCAGGGATTATCTAGATTGGGCAAAACTCTTTATATCCGATAAAAAtGAGACC-3´
3´-CCAGAGtTCACTCTTAGATCTAGTTTTCTCGTCCCTAATAGATCTAACCCGTTTTGAGAAATATAGGCTATTTTTaCTCTGG-5´
```

Pstrong10k

```
5´-GGTCTCaAGTGCCGGCATACCTCATCAACCCATTATTGGTAATTTTGGTCGCCGGGTGCTATATTGCACCAAAAAtGAGACC-3´
3´-CCAGAGtTCACGGCCGTATGGAGTAGTTGGGTAATAACCATTAAAACCAGCGGCCCACGATATAACGTGGTTTTTaCTCTGG-5´
```

Pstrong100k

```
5´-GGTCTCaAGTGCTAGCTATCTACATATATATATATGTTGACAGGGGCCATACATAATGCTATAATGCTGGGAAAAtGAGACC-3´
3´-CCAGAGtTCACGATCGATAGATGTATATATATATACAACTGTCCCCGGTATGTATTACGATATTACGACCCTTTTaCTCTGG-5´
```

Figure S2: Sequences of the three promoters generated with the promoter generator (https://salislab.net/software/). Red boxes show the BsaI recognition sites of the lvl 0 modules carrying the promoters, red dotted lines show the overhangs generated by BsaI restriction, blue boxes show the -35 element while purple boxes show the -10 element. Annotations were taken from the SalisLab Software. The deletion of an A in Pstrong100k is shown through a clear box with a red frame.*

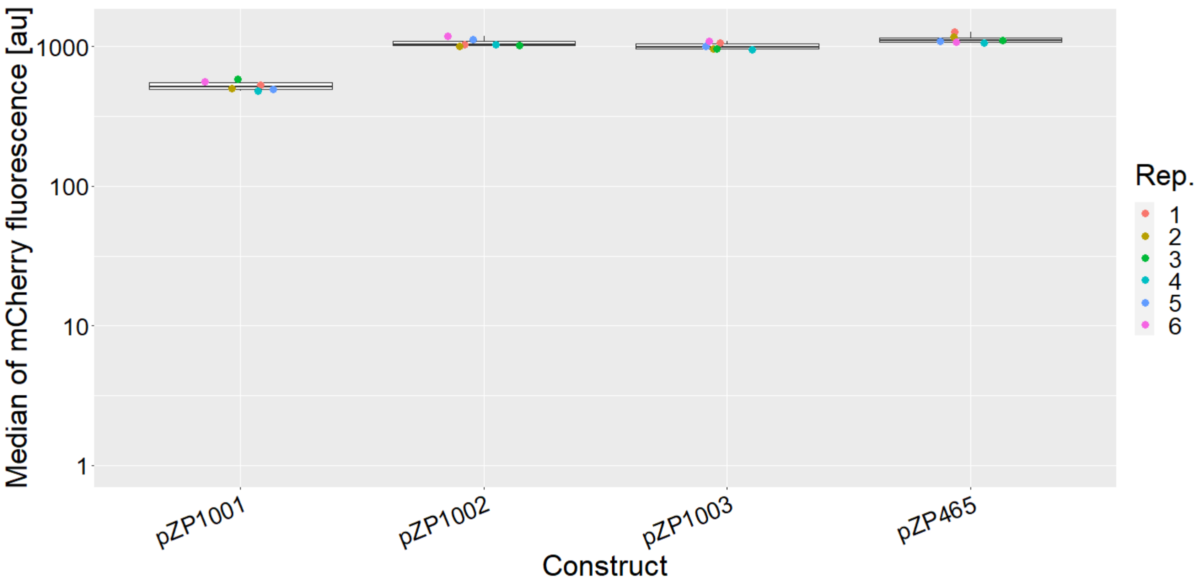

Figure S3: Boxplots showing the medians of mCherry fluorescence in ZM4 with plasmids pZP465, pZP1001, pZP1002 and pZP1003. The plasmid differ only in the terminator used: pZP465 – TsoxR, pZP1001 - TrrnB1, pZP1002 - TaspA, pZP1003 - TrplM-rpsI. In total six biological replicates (Rep.) from two independent cultivations were measured using flow cytometry.

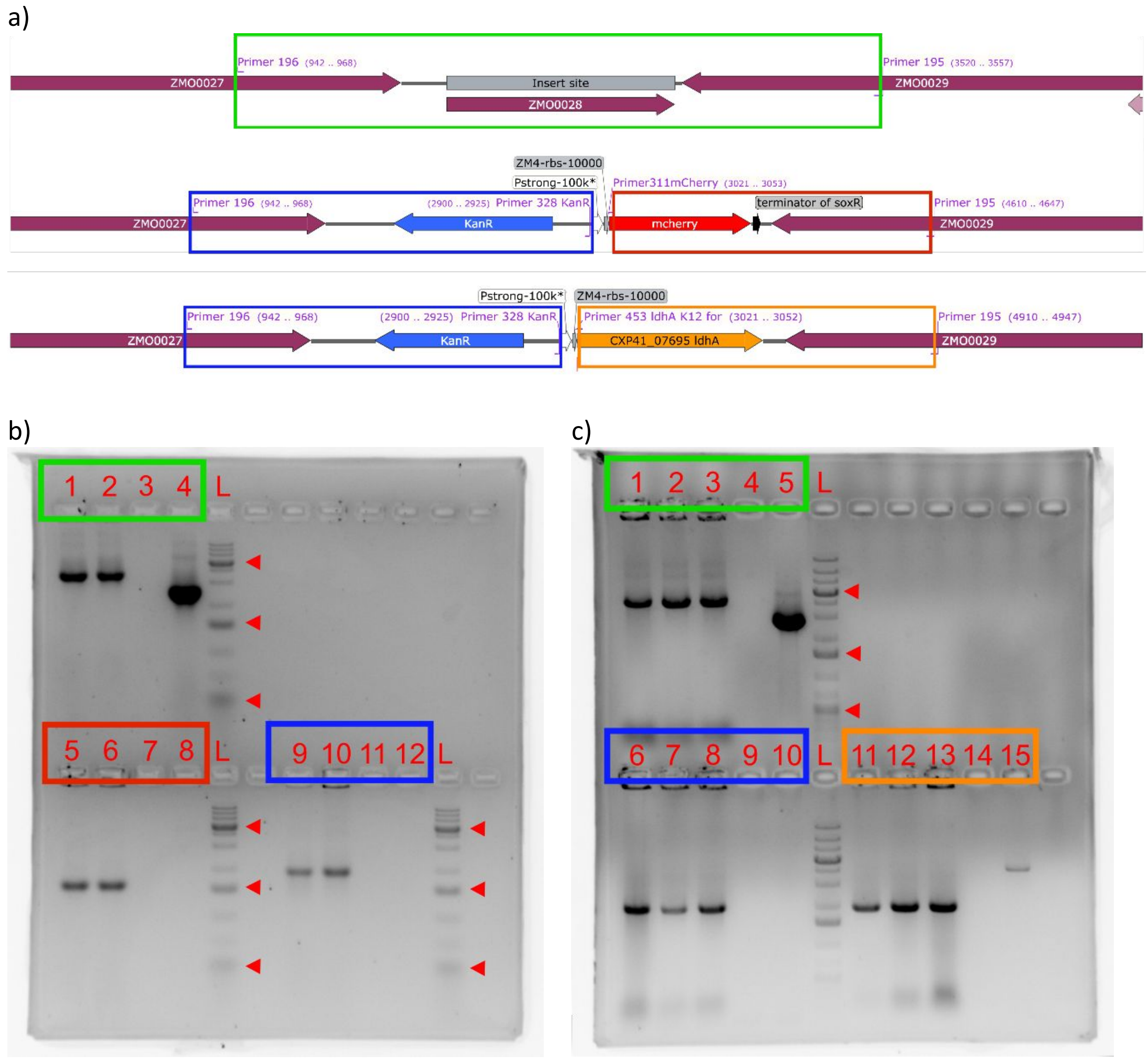

Figure S4: PCR proving the insertion of the mcherry TU and Kan^R into the chromosomal locus ZMO0028.
a) Maps of the wild-type locus (top), the edited locus that is created by insertion of Kan^R and an expression unit for mcherry (middle) into the chromosome using pZP778 and the edited locus created by insertion of Kan^R and an expression unit for ldhA into the chromosome using pZP1013. The binding sites of primers 195, 196, 311,328 and 453 are shown in pink. Primers 195 and 196 were used for the amplification of the whole locus (3706 bp in the edited mcherry version, 4006 bp in the edited ldhA version and 2616 bp for the wild-type, green box), both primers bind outside of the homology arms so that they could not amplify the region on pZP778 or pZP1013. Primers 195 and 311 amplify the region spanning from mcherry to the bordering locus ZMO0029 (amplificate of 1627 bp, red box). Primers 196 and 328 amplify the region spanning from Kan^R to the neighboring locus ZMO0027 (amplificate of 1984 bp, blue box). Primers 453 and 195 amplify the region spanning from ldhA to the bordering locus ZMO0029 (amplificate of 1927 bp, orange box).
b) shows the resulting PCR amplificates controlling the chromosomal insertion through pZP778 separated by agarose gel electrophoresis. L shows the DNA ladder GeneRuler 1 kb Plus (ThermoFisher Scientific), the red arrows indicate DNA sizes of 5000, 1500 and 500 bp. Lanes 1 to 4 show the PCR products generated using primers 195 and 196. Lanes 1 and 2 are edited ZM4 strains, 3 is a no template control and 4 used purified DNA of wild-type ZM4 as a template. Lanes 5 to 8 show the PCR products generated with primers 195 and 311. Lanes 5 and 6 are again the same edited ZM4 strains, while 7 is a no template control and 8 used purified DNA of wild-type ZM4 as a template. Finally, lanes 9 to 12 show the PCR products using primers 196 and 328, lanes 9 and 10 are the same representatives of the edited ZM4, while lane 11 shows a control with no template used and lane 12 had purified DNA of wild-type ZM4 as PCR template. Lanes 3, 7 and 11 are no template controls. Lanes 8 and 12 with purified wild-type DNA give no PCR products either, as only strains with the specific insert should generate amplificates, lane 4 shows a band around 2600 bp, which would match the expected wildtype amplificate.
c) shows the resulting PCR amplificates controlling the chromosomal insertion through pZP1013 separated by agarose gel electrophoresis. L shows the DNA ladder GeneRuler 1 kb Plus (ThermoFisher Scientific), the red arrows indicate DNA sizes of 5000, 1500 and 500 bp. Lanes 1 to 5 show the PCR products generated using primers 195 and 196. Lanes 1, 2 and 3 are edited ZM4 strains, 4 is a no template control and 5 used purified DNA of wild-type ZM4 as a template. Lanes 6 to 10 show the PCR products generated with primers 196 and 328. Lanes 6, 7 and 8 are again the same edited ZM4 strains, while 9 is a no template

control and 10 used purified DNA of wild-type ZM4 as a template. Finally, lanes 11 to 15 show the PCR products using primers 195 and 453, lanes 11, 12 and 13 are the same representatives of the edited ZM4, while lane 14 shows a control with no template used and lane 15 had purified DNA of wild-type ZM4 as PCR template. Lanes 4, 9 and 15 are no template controls. Lane 10 with purified wild-type DNA gives no PCR products either, as only strains with the specific insert should generate amplificates, lane 5 shows a band around 2600 bp, which would match the expected wildtype amplificate, lane 15 shows a faint band around 4000 bp which might be a random amplificate generated by the primers binding somewhere else in the genome, it is clearly different from the expected band for the insert at 2000 bp.

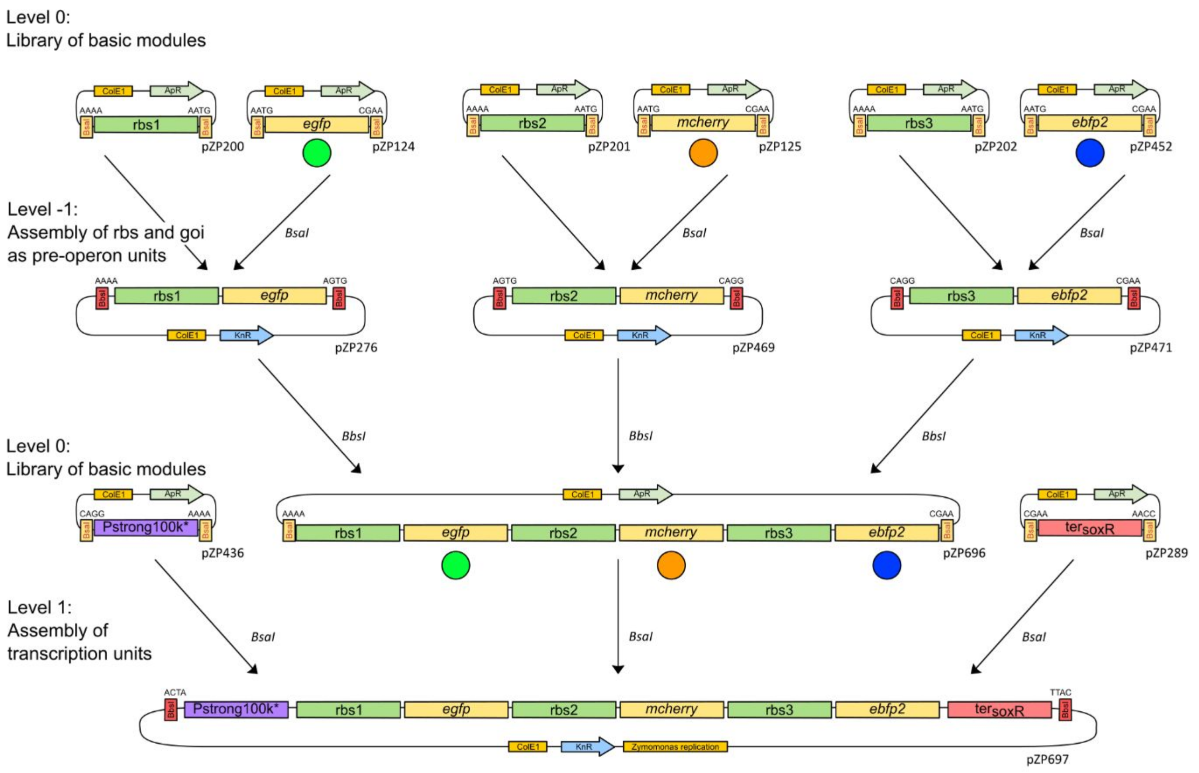

Figure S5: Graphical overview of the construction of plasmid pZP697 for the expression of the three fluorescence genes egfp, mcherry and ebfp2 in an operon.

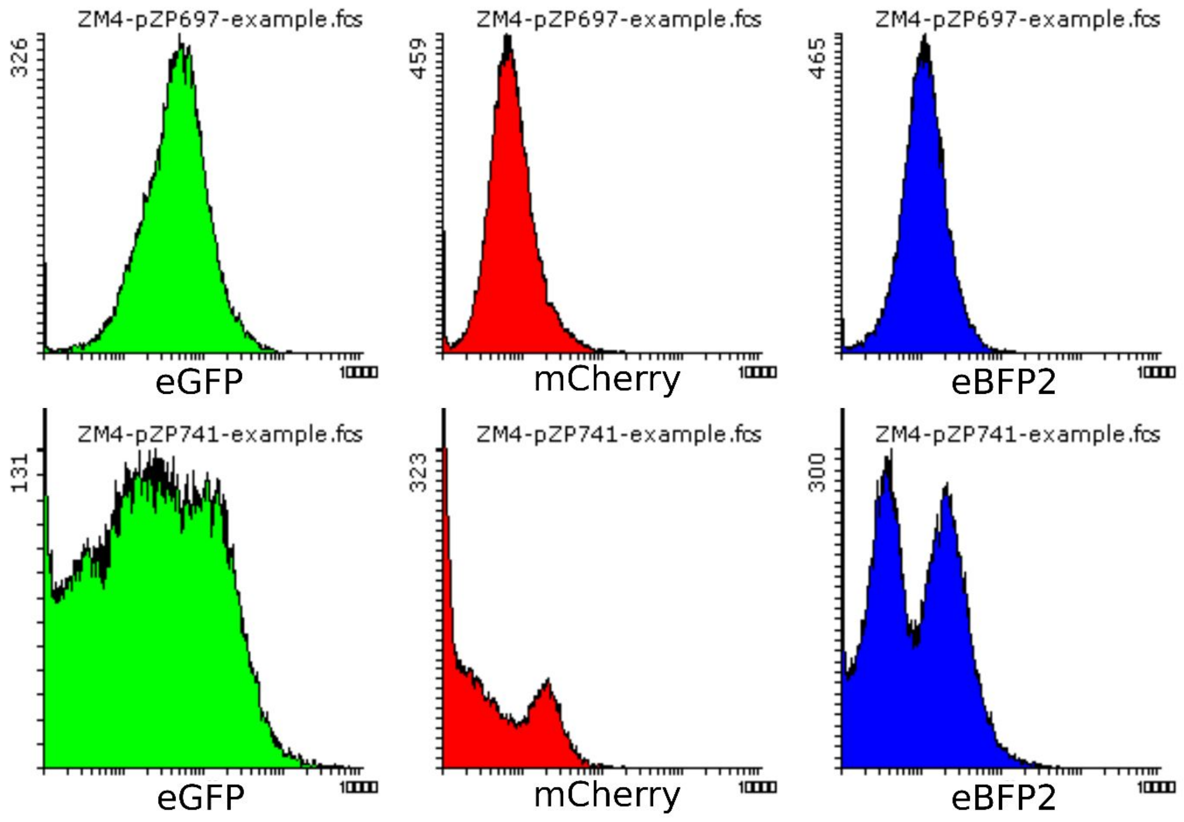

Figure S6: Histograms of fluorescence measurements by flow cytometry. The top row shows singular fluorescence peaks generated by pZP697, where different rbs were inserted in front of the different genes. The bottom row shows fluorescence distributions generated by pZP741, which has the same rbs in front of each gene, generating three 33 bp homologous regions, which most likely cause recombination of the plasmid in Z. mobilis. The abscissa shows the intensity of fluorescence for each fluorescent protein in artificial units and the ordinate the number of counts per intensity.

a)

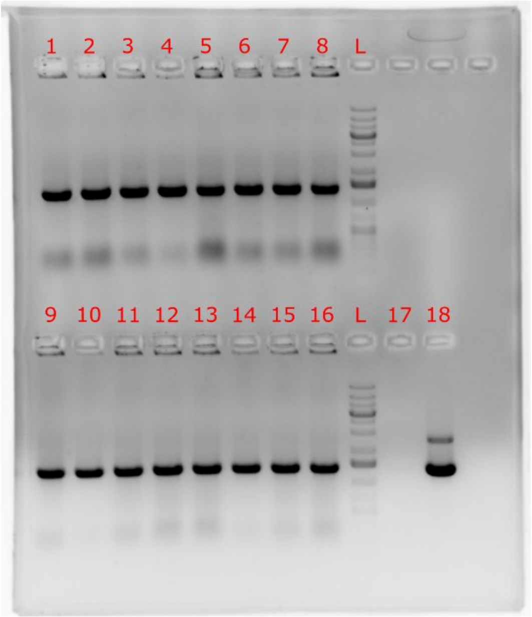

b)

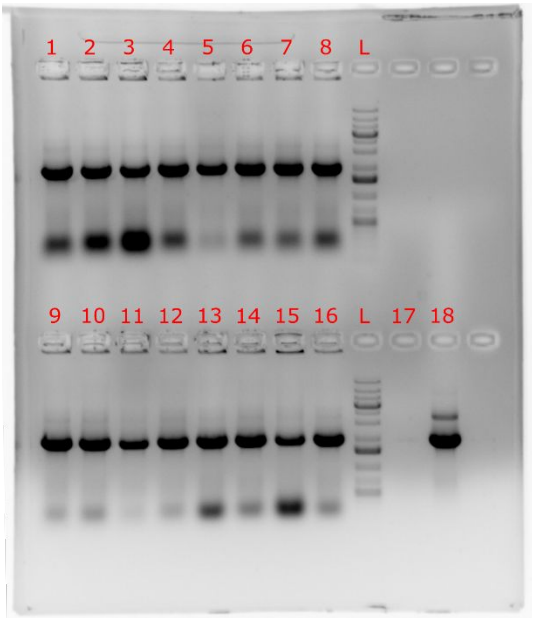

c)

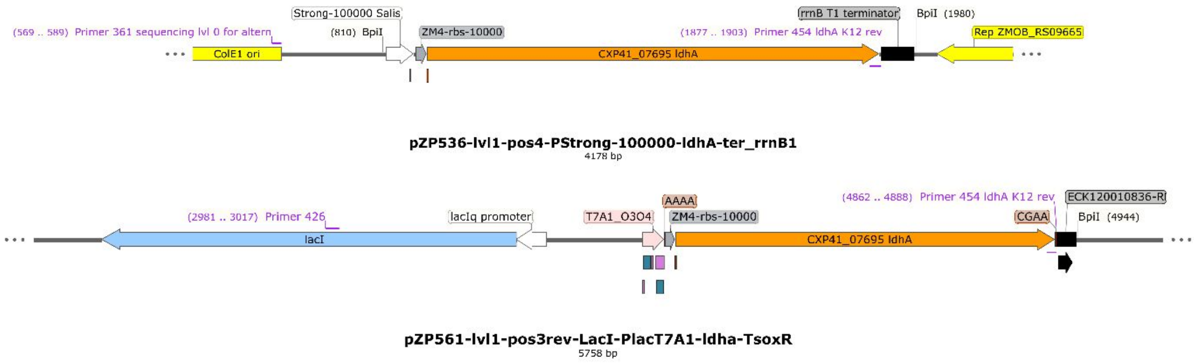

Figure S7: PCR control of colonies of pZP536 (a) and pZP561 (b). In both gels, lanes 1 to 16 show PCR reactions performed on colonies number 1 to 16, while lane 17 shows a no template control and lane 18 shows a positive control using 50 ng of plasmid pZP536 or pZP561 as template. L shows the DNA ladder GeneRuler 1 kb Plus (ThermoFisher Scientific). The sizes of the amplificates were expected to be 1335 bp and 1908 bp for pZP536 and pZP561, respectively, which fits well to the bandspresent. The bands over the amplificates in the positive control correspond to the plasmid used as template. c) shows maps of the plasmids pZP536 and pZP561 with primers used for the qualitative PCR indicated in pink (primers 361 and 454 for pZP536 and primers 426 and 454 for pZP561).

Table S1: Overview of lactate production: ni (not induced), nd (not determined), const. (constitutive expression)

		Biomass		Glucose		Ethanol			Lactate		
Strain	Induction	Growth rate 1/h	Yield coefficient (g CDW/ mol Glc)	Specific uptake (mmol/ CDW*h)	Initial titer (mM)	Specific production (mmol/ CDW*h)	Final titer (mM)	Yield (mol/ mol Glc)	Specific production (mmol/ CDW*h)	Final titer (mM)	Yield (mol/ mol Glc)
WT	ni	0.225	4.16	54.50	204.7 ± 7.8	102.90	383.8 ± 5.0	1.87	0	0	0
pZP537 EV	const.	nd	4.66	nd	209.0 ± 0.9	nd	376.8 ± 4.2	1.80	0	0	0
pZP561	ni	nd	4.17	nd	216.7 ± 6.1	nd	353.6 ± 5.2	1.63	nd	19.6 ± 0.6	0.09
	10 µM IPTG	nd	4.22	nd	221.5 ± 2.7	nd	326.7 ± 7.2	1.48	nd	42.3 ± 4.8	0.19
	100 µM IPTG	0.219	3.92	51.49	217.2 ± 3.2	73.64	294.1 ± 3.5	1.35	19.01	70.6 ± 3.7	0.33
	500 µM IPTG	0.212	3.66	47.67	219.2 ± 7.8	62.74	282.6 ± 2.3	1.29	21.24	85.2 ± 2.4	0.40
	1 mM IPTG	nd	3.90	nd	216.0 ± 16.0	nd	255.9 ± 2.4	1.18	nd	83.1 ± 0.9	0.38
pZP255	216 nM aTc	0.241	3.76	68.74	210.6 ± 7.7	89.25	382.2 ± 2.8	1.81	15.47	35.1 ± 1.7	0.17
	500 nM aTc	0.191	4.10	44.78	203.9 ± 7.8	84.61	371.0 ± 4.2	1.81	6.05	29.0 ± 1.9	0.14
pZP374	const.	0.195	4.73	46.98	202.3 ± 3.4	68.74	305.7 ± 2.8	1.51	21.19	78.4 ± 2.6	0.38
pZP536	const.	nd	4.84	nd	214.9 ± 6.2	nd	238.3 ± 5.7	1.11	nd	85.2 ± 4	0.41
ΔZMO002 ::*ldhA*	const.	0.200	4.43	48.25	205.1 ± 1.0	79.31	327.4 ± 5.2	1.60	10.14	39.7 ± 0.6	0.21

Table S2: Colony counts for exemplary assessment of assembly efficiency of Zymo Parts. pZP536 and pZP561 are transcription units for ldhA in level 1 with different promoters, while pZP1001, pZP1002 and pZP1003 are transcription units for mcherry with different terminators in level 1.

Plasmid	Replicate	Volume	Number of col.	Number Red col.	Number Blue col.	Number White col.	Ratio Red:total	Ratio Blue:total	Ratio White:total
pZP536	1	10 µL	14	0	0	14		0.000	1.000
pZP536	1	50 µL	382	0	0	382		0.000	1.000
pZP536	2	10 µL	55	0	0	55		0.000	1.000
pZP536	2	50 µL	170	0	1	169		0.006	0.994
pZP536	3	10 µL	21	0	0	21		0.000	1.000
pZP536	3	50 µL	143	0	0	143		0.000	1.000
pZP561	1	10 µL	23	0	0	23		0.000	1.000
pZP561	1	50 µL	297	0	1	296		0.003	0.997
pZP561	2	10 µL	60	0	1	59		0.017	0.983
pZP561	2	50 µL	610	0	9	601		0.015	0.985
pZP561	3	10 µL	22	0	0	22		0.000	1.000
pZP561	3	50 µL	294	0	5	289		0.017	0.983
pZP1001	1	10 µL	41	38	2	1	0.927	0.049	0.024
pZP1001	1	50 µL	433	393	26	14	0.908	0.060	0.032
pZP1001	2	10 µL	31	29	0	2	0.935	0.000	0.065
pZP1001	2	50 µL	676	600	52	24	0.888	0.077	0.036
pZP1001	3	10 µL	83	76	6	1	0.916	0.072	0.012
pZP1001	3	50 µL	567	506	41	20	0.892	0.072	0.035
pZP1002	1	10 µL	25	23	2	0	0.920	0.080	0.000
pZP1002	1	50 µL	190	178	9	3	0.937	0.047	0.016
pZP1002	2	10 µL	13	11	1	1	0.846	0.077	0.077
pZP1002	2	50 µL	218	208	6	4	0.954	0.028	0.018
pZP1002	3	10 µL	26	24	1	1	0.923	0.038	0.038
pZP1002	3	50 µL	226	205	12	9	0.907	0.053	0.040
pZP1003	1	10 µL	26	25	0	1	0.962	0.000	0.038
pZP1003	1	50 µL	226	205	7	14	0.907	0.031	0.062
pZP1003	2	10 µL	23	23	0	0	1.000	0.000	0.000
pZP1003	2	50 µL	234	209	11	14	0.893	0.047	0.060
pZP1003	3	10 µL	30	26	3	1	0.867	0.100	0.033
pZP1003	3	50 µL	195	181	8	6	0.928	0.041	0.031
